# Supplementary material for: Foreflipper and hindflipper muscle reconstructions of Cryptoclidus eurymerus in comparison to functional analogues: introduction of a myological mechanism for flipper twisting
Source: PeerJ. 2021 Dec 15;9:e12537. doi: 10.7717/peerj.12537 (PMC8684327; doi:10.7717/peerj.12537)
Supplement: Supplemental Information 1 [file peerj-09-12537-s001.pdf]

Homology of plesiosaur foreflipper and hindflipper myology

Foreflipper

Musculus pectoralis

pectoralis (Watson, 1924; Tarlo, 1958; Robinson, 1975; Lingham-Soliar, 2000; Carpenter et al., 2010; Araújo & Correia, 2015)

Musculus subcoracoscapularis

subcoracoscapularis (Lingham-Soliar, 2000; Araújo & Correia, 2015)
subscapularis and subcoracoideus (Watson, 1924; Tarlo, 1958)
subscapularis, subcoracoideus, subcoracoscapularis (Robinson, 1975)
subscapularis and subclavicularis (Carpenter et al., 2010) (termed by position, but not really explained how Carpenter et al. (2010) came to this conclusion)

Musculus supracoracoideus

supracoracoideus (Watson, 1924; Tarlo, 1958; Robinson, 1975; Lingham-Soliar, 2000; Araújo & Correia, 2015)
supraclavicularis (Carpenter et al., 2010) (termed by Carpenter et al. (2010) by position but did not describe detailed how they came to the conclusion, so we presume it is supposed to be the supracoracoideus that has just been shifted anteriorly

Musculus coracobrachialis longus

coracobrachialis longus (Lingham-Soliar, 2000; Araújo & Correia, 2015)
coracobrachialia (Tarlo, 1958)
coraco-brachialis longus (Watson, 1924)
coracobrachialis (Carpenter et al., 2010), it is not possible to determine whether the coracobrachialis of Carpenter et al. (2010) is supposed to represent both muscular heads or only one subportion, but since it is separated into two heads in extant lepidosaurs and turtles (Walker, 1973; Russell & Bauer, 2008) on which their study is based, we presume that they imply by „coracobrachialis“ both, coracobrachialis brevis and longus.

Musculus coracobrachialis brevis

coracobrachialis brevis (*Lingham-Soliar*, 2000; *Araújo & Correia*, 2015)
coracobrachialis (Robinson, 1975; Carpenter et al., 2010)

coracobrachialia (Tarlo, 1958)
coraco-brachialis brevis (Watson, 1924)
coracobrachialis (Carpenter et al., 2010) (s. coracobrachialis longus)

Musculus scapulohumeralis anterior
scapulohumeralis anterior (Tarlo, 1958; Robinson, 1975; Lingham-Soliar, 2000)
scapulo-humeralis anterior (Watson, 1924)
scapulohumeralis (Carpenter et al., 2010) cannot surely be determined if they mean scapulohumeralis anterior or posterior, as they base their reconstructions on lepidosaurs, but also on *Sphenodon*, which has both muscles, but the position in which it is reconstructed hint to a scapulohumeralis anterior, but the homology remains doubtful

Musculus scapulohumeralis posterior
scapulohumeralis posterior (Lingham-Soliar, 2000)
scapulohumeralis (Araújo & Correia, 2015) as they synonymized it with the respective muscles in crocodilians and lepidosaurs

Musculus deltoideus clavicularis
deltoideus clavicularis (Carpenter et al., 2010)
deltoides clavicularis (Robinson, 1975; Lingham-Soliar, 2000)
clavodeltoideus (Araújo & Correia, 2015)
deltoid (Watson, 1924), as this muscle was reconstructed to take its origin from the clavicula and the scapula, it is probable that Watson (1924) means both, deltoideus scapularis and deltoideus clavicularis with this term, which is substantiated by Fig. 5, p. 896 in which a humeral „Del Sc“ and „Del Cl“ are marked, but the authors could not find a legend to these abbreviations in Watson (1924).

Musculus deltoideus scapularis
deltoideus scapularis (Carpenter et al., 2010), abbreviation of this muscle listed in Fig.17, p. 22 but not pictured, but instead the deltoideus clavicularis is shown to arise probably from the scapular prong, which is rather the origin surface for the deltoideus scapularis
scapulodeltoideus (Araújo & Correia, 2015)
deltoides scapularis (Robinson, 1975; Lingham-Soliar, 2000)
scapular deltoid (Tarlo, 1958)

deltoid (Watson, 1924), please view comment on this in „deltoideus clavicularis“ section

Musculus latissimus dorsi

latissimus dorsi (Watson, 1924; Tarlo, 1958; Robinson, 1975; Lingham-Soliar, 2000; Carpenter et al., 2010; Araújo & Correia, 2015)

teres major (Carpenter et al., 2010)

Musculus triceps brachii

triceps (Robinson, 1975)

triceps brachii (Araújo & Correia, 2015)

Musculus biceps brachii

biceps (Robinson, 1975; Carpenter et al., 2010)

biceps brachii (Araújo & Correia, 2015)

Musculus brachialis

brachialis (Robinson, 1975)

Musculus flexor carpi ulnaris

flexor carpi ulnaris (Robinson, 1975)

Musculus flexor carpi radialis

flexor carpi radialis (Robinson, 1975)

Hindflipper

Musculus iliotibialis

iliotibialis (Robinson, 1975; Lingham-Soliar, 2000)

Musculus femorotibialis

femorotibialis (Robinson, 1975)

Musculus ambiens

ambiens (Robinson, 1975)

Musculus ischiotrochantericus

ischiotrochantericus (Robinson, 1975)

Musculus iliofibularis

iliofibularis (Robinson, 1975; Lingham-Soliar, 2000)

Musculus puboischiotibialis

puboischiotibialis (Robinson, 1975; Lingham-Soliar, 2000)

Musculus adductor femoris

adductores (Robinson, 1975)

„Primitively the adductor arises midventrally from the puboischiadic ligament…“ (Robinson, 1975). In lepidosaurs adductor femoris originates from the puboischiadic ligament (Snyder, 1954) and this seems to fit well name-wise as well as in terms of described insertion area on the femur. In turtles fti, pubtib, pubischtib originate from the aforementioned ligament, but they span the femur (Walker, 1973), so adductores cannot be one of them. Crocodilians have no puboischiadic ligament, so they provide no further information here.

Musculus caudifemoralis brevis

caudifemoralis brevis (Robinson, 1975)

caudofemoralis (Carpenter et al., 2010), not clearly deducible whether or not it represents both muscular heads of caudifemoralis or simply brevis. Fig. 18 A and 14 D rather suggest that they only refer to caudifemoralis brevis, although in lepidosaurs c. brevis and longus is present and only in turtles there is only one head, c. brevis

caudifemoralis (Lingham-Soliar, 2000) (seems to be synonymous to both portions, (s. Fig. 10 D) because two muscle bellies are shown of which one runs posterior/caudal to the tail)

Musculus caudifemoralis longus

caudifemoralis longus (Robinson, 1975)

caudifemoralis (Lingham-Soliar, 2000)

Musculus puboischiofemoralis externus

puboischiofemoralis externus (Robinson, 1975; Lingham-Soliar, 2000; Carpenter et al., 2010)
ischiofemoralis externus (Carpenter et al., 2010), difficult to determine which muscle this is supposed to be in turtles and lepidosaurs, but given its origin area on the pelvic girdle it seems to be reasonable to presume that this is an ischial head of puboischiofemoralis externus (compare to Snyder (1954), Walker (1973), Russell & Bauer (2008)) as it is also shown to insert together with the PIFE into the femur (compare Fig. 17 F, Carpenter et al. (2010))

Musculus puboischiofemoralis internus

puboischiofemoralis internus (Robinson, 1975; Lingham-Soliar, 2000; Carpenter et al., 2010)
pubofemoralis internus (Lingham-Soliar, 2000)
ischiofemoralis internus (Carpenter et al., 2010), it is difficult to determine which muscle this is supposed to be as the authors do not describe how they have come to the conclusion. Given its position on the pelvic girdle, this muscle could be an ischial head of puboischiofemoralis internus, which would only be supported by lepidosaurs (and not turtles, as their EPB is based on these two taxa). A separated insertion into the femur for Pifi (as shown in Fig. 17 E, Carpenter et al. (2010)) is supported by Russell & Bauer (2008), although Snyder (1954) depicts a common insertion as shown for turtles, too (Walker, 1973).
Supported by turtles and lepidosaurs would be ischiotrochantericus, which would also be in agreement with Fig. 17 E by Carpenter et al. (2010), in which this muscle is shown to insert independently from PIFI. Yet, its anterior position on the ischium is only supported by turtles, not by lepidosaurs, in which pifi arises from this position. So both muscles are thinkable to be synonymous to ischiofemoralis internus defined by their origin areas. The part of the name „internus“ might hint, that it is supposed to be a portion of PIFI though.

Musculus iliofemoralis

iliofemoralis (Robinson, 1975; Lingham-Soliar, 2000; Carpenter et al., 2010)

Musculus peroneus brevis and Musculus peroneus longus

peronaeus longus (Robinson, 1975)

Musculus tibialis anterior

tibialis anticus (Robinson, 1975)

References

Araújo R, Correia F. 2015. Soft-tissue anatomy of the plesiosaur pectoral girdle inferred from basal Eosauropterygia taxa and the extant phylogenetic bracket. *Palaeontologia Electronica* **18 (1)**:1–32.

Carpenter K, Sanders F, Reed B, Reed J, Larson P. 2010. Plesiosaur swimming as interpreted from skeletal analysis and experimental results. *Transactions of the Kansas Academy of Science* **113 (1/2)**:1–34.

Lingham-Soliar T. 2000. Plesiosaur locomotion: Is the four-wing problem real or merely an atheoretical exercise? *Neues Jahrbuch für Geologie und Paläontologie, Abhandlungen* **217 (1)**:45–87.

Robinson JA. 1975. The locomotion of plesiosaurs. *Neues Jahrbuch für Geologie und Paläontologie, Abhandlungen* **149**:286–332.

Russell AP, Bauer AM. 2008. The appendicular locomotor apparatus of *Sphenodon* and normal-limbed squamates. In: Gans C, Parsons TS, editors. *Biology of the Reptilia* Volume 21 New York: Academic Press. 1–465.

Snyder RC. 1954. The anatomy and function of the pelvic girdle and hindlimb in lizard locomotion. *The American Journal of Anatomy* **95 (1)**:1–45.

Tarlo LB. 1958. The scapula of *Pliosaurus macromerus* Phillips. *Palaeontology* **1**:193–199.

Walker WF. 1973. 1973. The locomotor apparatus of Testudines. In: Gans C, Parsons TS, editors. *Biology of Reptilia* Volume 4. New York: Academic Press.1–100.

Watson DMS. 1924. The elasmosaurid shoulder-girdle and fore-limb. *Proceedings of the Zoological Society of London* **58**:885–917.
